# Contribution of IL-33 to the Pathogenesis of Colorectal Cancer

**DOI:** 10.3389/fonc.2018.00561

**Published:** 2018-11-28

**Authors:** Guanglin Cui, Aping Yuan, Zhigang Pang, Wei Zheng, Zhenfeng Li, Rasmus Goll

**Affiliations:** ^1^Research Group of Gastrointestinal Diseases, The Second Affiliated Hospital of Zhengzhou University, Zhengzhou, China; ^2^Faculty of Health Science, Nord University, Levanger, Norway; ^3^Department of Gastroenterology, University Hospital of North Norway, Tromsø, Norway

**Keywords:** colorectum, tumorigenesis, cytokine, IL-33, ST2

## Abstract

The development of colorectal cancer (CRC) is not only determined by transformed cells *per se*, but also by factors existing in their immune microenvironment. Accumulating scientific evidence has revealed that interleukin (IL)-33, an IL-1 family member, plays an essential role in the regulation of immune response and is relevant in CRC pathogenesis. Data from both human and experimental studies demonstrated that IL-33 inhibits host anti-tumor immunity, remodels tumor stroma and enhances angiogenesis, thereby promoting the development of CRC. These pro-tumor effects of IL-33 are mainly mediated by IL-33 receptor ST2 (also known as IL-1RL1). Based on those findings, it is currently hypothesized that the IL-33/ST2 pathway is a potential biomarker and therapeutic target for colorectal tumorigenesis. Herein, we summarize the recent discoveries in understanding the critical role of the IL-33/ST2 pathway in contributing to the pathogenesis of colorectal tumorigenesis and discuss its potential implications for the future development of effective anti-tumor strategies.

## Introduction

Colorectal cancer (CRC) is a leading cause of cancers mortality worldwide. The initiation and development of CRC is not only determined by the genetic and molecular characteristics of transformed cells *per se*, but also by factors existing in their immune microenvironment (1–3).

Host immune function is one of the most important factors contributing to the development of CRC (4–6). The interaction between host immunity and transformed cells might significantly influence the establishment of CRC (1, 2, 7). It has been known for a number of years that there is a significant impairment of host anti-tumor immunity during the initiation of CRC because transformed cells, in order to escape host immune attack, use diverse strategies to suppress anti-tumor immunity and form a favorable growth milieu. Therefore, there is great interest in studying the contributing factors of immune microenvironment formation in premalignant and malignant lesions.

The lack of an effective immune response to tumorigenesis, in spite of the presence of dense tumor infiltrating lymphocytes in the tumor microenvironment, is believed to be due in some way to the action of immunosuppressive immune cells/cytokines (8–10). Certain cytokines can significantly affect the initiation/progression of CRC (1, 11–14), acting not only as the promotors for tumor cell growth but also as important contributor for the immune suppressive microenvironment formation during tumor development (4, 15–25). Therefore, the improved characterization of cytokine networks will greatly add help in understanding the cross-talk between transformed cells and their microenvironment, thereby presenting the prospect of designing novel translational/targeted approaches to tumors (14, 26–28). In the study of cytokine networks in the colorectal tumorigenesis, a greatly altered cytokine profile during progression of adenomas to CRCs has been demonstrated (14, 18, 29–31), in which the level of Th1 cytokines is decreased but the expression level of Th2 cytokines is significantly increased from adenomas to CRCs (5, 6, 32, 33). Further immunohistochemical analysis demonstrated that adenoma/CRC epithelium significantly contributes to the production of cytokines (5, 20, 23, 34–38), whereas, blocking cytokine signaling may suppress the development and establishment of both premalignant and malignant lesions in animal models (39, 40). Numerous studies strongly suggest that cytokines are key factors in influencing the development of CRC (2, 15, 35, 41, 42).

IL-33 is a novel pro-inflammatory cytokine that belongs to the IL-1 family. IL-33 plays an important role in the regulation of host immune responses (43–45) and has been associated with human inflammatory bowel diseases (IBD) (46, 47), as well as tumors (48–55). Moreover, studies have also revealed that high expression of IL-33 is associated with poor prognosis in diverse cancers and may be a potential prognostic predicator (52, 54–58). Current data strongly suggest that IL-33 is involved in the pathogenesis of CRC (19, 59, 60).

In view of the importance of IL-33 in contributing to the pathogenesis of CRC, herein, we review the critical role of IL-33 in the pathogenesis of CRC development and evaluate its potential as biomarker for assessing tumor aggressiveness and disease prognosis. In addition, the possible role of IL-33 and its receptor, ST2, as novel biomarkers and therapeutic targets is discussed.

## Cells expressing IL-33 and its functional receptor, ST2, in the tumor microenvironment

We previously examined cell expressing IL-33 and ST2 in the human adenoma/CRC microenvironment. Results showed that IL-33 immunoreactivity (IR) is observed in diverse cells, including adenoma/CRC cells, CD3 positive lymphocytes, SMA-alpha positive myofibroblasts and CD34 positive endothelial cells of tumor-associated microvessels (19). IL-33 functions as a pro-inflammatory cytokine by binding to the heterodimeric ST2 receptor complex (46). Interestingly, ST2 in the adenoma/CRC microenvironment is expressed in a very similar cellular pattern as IL-33. ST2-IR has been clearly identified in adenoma/CRC cells, lymphocytes, myofibroblasts and endothelial cells of microvessels (19). Those observations indicate that IL-33 and ST2 within the adenoma/CRC microenvironment originate from a mixed cellular source and provide important information for understanding the interaction between IL-33 and its receptor, ST2, in promoting colorectal tumorigenesis.

## Promoting effects of IL-33 on the development of colorectal tumorigenesis

Chronic inflammation is a major risk factor for the development of CRC (1, 15, 61), and IL-33 is a potent pro-inflammatory cytokine that plays a critical role in the initiation of colorectal mucosal inflammation (62, 63). Thus, studies regarding the role of IL-33 in the CRC development in animal models has attracted much attention and evidence is accumulating.

The one animal study of interest with respect to IL-33 and the formation of premalignant adenoma was performed in mice. Maywald et al. examined the promoting effect of IL-33 in the formation of polyps (adenomas) in traditional ApcMin/+ mice with an IL-33 deficient background (59). They found that IL-33 deficiency significantly reduced the formation of intestinal polyposis (adenoma) and mast cell accumulation in adenomatous polyps, suppressing the expression of mast cell-derived proteases and cytokines known to promote polyposis in ApcMin/+ mice (59). Thus, their findings suggest that IL-33 promotes the formation of intestinal adenomas through coordinated activation of stromal cells and formation of a pro-tumorigenic microenvironment (59).

We have examined the expression profile of IL-33 in human adenomas and found that expression levels of IL-33 mRNA are markedly increased in adenoma tissues (19). This finding might imply that IL-33 is an early immune response element reacting to the initiation of premalignant adenomas. Immunohistochemical results revealed that IL-33-IR was highly expressed in adenoma cells, lymphocytes, stromal cells (myofibroblasts) and microvessels, indicating an autocrine/paracrine loop in those cells. Particularly, the density of IL-33-positive microvessels was significantly increased in adenoma stroma compared to normal controls (19). These findings suggest that elevated IL-33 may participate in the regulation of tumor associated angiogenesis during the progression of adenomas. Indeed, we have found that the expression level of IL-33 mRNA is associated with dysplastic degree in adenomas (19). Those findings suggest the hypothesis that IL-33 participates in the pathogenesis of premalignant adenomas.

Regarding the role of IL-33 in the pathogenesis of CRC, numerous studies have been conducted in animals and humans.

Using mice treated with the chemical carcinogen azoxymethane (AOM) and the inflammatory inducer dextran sodium sulfate (DSS), Islam et al. revealed that epidermal growth factor is a key growth factor in intestinal epithelial cell (IEC)-derived IL-33 promotion of tumorigenesis (64). Most current human studies are performed in established human CRCs (19, 59, 63). Results have showed increased expression levels of IL-33 at both the protein and transcriptional levels in human CRC tissues (19). Interestingly, increased expression levels of IL-33 and ST2 were more frequently observed in low-grade adenocarcinomas than high-grade adenocarcinomas (63). Our immunohistochemical analysis has shown that many types of cells within the CRC microenvironment can express both IL-33 and ST2. As illustrated in adenomas, we also found that both IL-33- and ST2-IRs are expressed in CRC cells, CD3 positive lymphocytes, SMA-alpha positive myofibroblasts, and CD34 positive microvessels within the CRC microenvironment. Our findings suggest that elevated expression of IL-33 and ST2 in CRC tissues may come from a mixed cellular source, and IL-33 could have far-reaching biological effects on diverse cells within the CRC microenvironment.

## Contribution of different forms of the IL-33 receptor to the development of colorectal tumorigenesis

The IL-33 receptor exists in the following two forms as splice variants: (1) a soluble form (sST2), which acts as a decoy receptor, sequesters free IL-33, and does not signal; and (2) a membrane-bound form (ST2), which activates MyD88/NF-κB signaling to enhance mast cell, Th2, Treg cells, and innate lymphoid cell type 2 functions (65). Therefore, the functions for these two receptor forms are completely different. It has been reported that the expression level of ST2 is significantly increased in both animal and human CRC tissues (19, 63). Moreover, the administration of ST2 antibody in ApcMin/+ mice results in significantly reduced both tumor number and size compared to control mice, accompanied by a significant inhibition of proliferation and angiogenesis, with increased apoptosis in adenomatous polyps (59). Mertz KD and colleagues examined the role of ST2 in the development of colorectal tumorigenesis in an ST2–/– deficient mouse model (63). They found that deficiency of ST2 in mice results in a protective effect against AOM/DSS-triggered CRC, with significantly reduced tumor load compared with control mice. In addition, their results also suggested that the promoting effect of the IL-33/ST2 axis on colonic tumorigenesis occurs via the production of IL-6 (63). Their results strongly suggest that ST2 is a key mediator for IL-33 induced colorectal tumorigenesis (63). We examined the expression profile of ST2 in both human adenomas and CRCs (19). Results demonstrated that increased expression of ST2 is found in adenoma tissues and persists to in CRC tissues. ST2-IR is highly expressed in adenoma/CRC cells, stromal cells and microvessels within the tumor microenvironment (19). Since ST2 plays an essential role in the regulation of host immune response, Treg accumulation, and angiogenesis, our findings indicates that increased expression of ST2 participates in the development of CRC.

The function of sST2 is different from that of ST2, and the role of sST2 during the progression of CRC has also been examined. One study revealed that the expression level of sST2 in cancer tissues is lower than that in adjacent nontumor tissues. In addition, patients with advanced TNM stage have lower expression of sST2 (66). They only found differences in ST2, sST2 was only measured in plasma with no difference between patients *vs*. healthy subjects. Which suggests that the ST2 variant might have an anti-tumorigenic role in the CRC. In contrast to O'Donnell's results (66), Miho Akimoto et al. reported that sST2 is a negative regulator for tumor growth and metastasis (67). They found that expression levels of sST2 are inversely associated with the malignant growth of CRC, and sST2 is down regulated in highly metastatic cells compared with low metastatic human and mouse CRC cells. Knockdown of sST2 in low metastatic SW480 cells (CRC cell line derived from the primary tumor site) with sST2 short hairpin RNA (shRNA) enhances tumor cell growth and tumor formation, whereas its overexpression in highly metastatic SW620 cells (CRC cell line derived from the metastatic site from the same patient) inhibits these processes. The SW480-shsST2 cells efficiently formed tumors (7 out of 7 mice), whereas the SW480-shCont (the control shRNA-expressing) cells seldom formed tumors (1 out of 7 mice). It has been previously shown that IL-33 exhibited a proangiogenic effect in human umbilical vein endothelial cells (HUVECs) via Akt signaling (68). The authors have therefore examined the ratio of phospho-Akt/total Akt in HUVECs treated with rIL-33 alone or in combination with rsST2. They found that rIL-33 promoted Akt phosphorylation, and phosphorylation was decreased by co-treatment with rsST2. Similarly, a thymidine incorporation assay, migration assay and tube formation assay using a two-dimensional Matrigel revealed the inhibitory effect of rsST2 on rIL-33-induced responses of HUVECs (67). Thus, they concluded that sST2 is involved in the regulation of IL-33 on angiogenesis. These two reports have provided different results. Therefore, further studies regarding the exact role of sST2 in CRC progression still needed.

Collectively, these results obtained from both animal and human studies suggest that colorectal tumorigenesis elicits a strong IL-33 response, and this response may contribute to the development of both premalignant and malignant lesions.

## Mechanisms of IL-33 promoting the development of CRC

The precise mechanisms whereby IL-33 promotes the development of CRC remain unclear, though several potential mechanisms have been hypothesized and evaluated.

A direct effect on colorectal epithelial cells: A direct promoting effect of IL-33 on cell transformation has been observed in other types of human cancers (49). However, Mertz KD et al. revealed that the promoting effect of IL-33 on the development of CRC in mice does not directly affect the proliferation of tumor cells in animal CRC models (63).

Induction of other pro-tumorigenic factors: Cyclooxygenase-2 (COX-2), IL-6 and IL-17A are potent pro-tumorigenic factors that encourage the development of CRCs (22, 24, 30, 69, 70). It was recently shown that the stimulating effect of IL-33 on cell proliferation *in vitro* with primary CRC cells, as well as CRC cell lines, depends on activation of the COX-2/PGE2 pathway (71). The COX-2 selective inhibitor and PGE2 neutralizing antibody significantly attenuated such proliferation-promoting effects induced by IL-33 administration (71). IL-17A is another pro-inflammatory cytokine with a strong pro-tumorigenic capacity. Studies have also shown that serum concentrations of IL-33 are associated with upregulated levels of IL-17A in patients with autoimmune hepatitis, whereas specific inhibition of IL-33 by treatment with a specific neutralizing antibody significantly reversed the level of IL-17A in a murine model with autoimmune hepatitis (72). Moreover, a recent study demonstrated that IEC-derived IL-33 regulates the accumulation of Th17 cells (a main cellular source of IL-17A) in the small intestine (73). In addition, IL-6 is also reported to be an important mediator for IL-33 in promoting the formation of CRC in ST2 deficient mice (63). Since IL-6 is an upstream stimulator of IL-17A production from Th17 cells, the IL-33-IL-6-IL-17A pathway may play a critical role in the development of CRC.

Remodeling tumor stroma and pro-angiogenesis: IL-33 markedly stimulates myofibroblasts to produce several types of extracellular matrix components including MMP2, MMP9, and growth factors associated with CRC tumor growth, progression and metastasis (63, 67, 74). Such stromal remodeling effects of IL-33 have been shown to promote tumor cell growth and liver metastasis in IL-33-deficient mice with a metastatic murine CRC cell line (60).

IL-33 is a cytokine with strong pro-angiogenic capabilities (68, 75, 76), and one of the potential mechanisms for IL-33 in promoting CRC progression maybe due to the enhanced angiogenesis (60). We know that the IL-33 receptor, ST2, is highly expressed in the endothelial cells of microvessels that mediate potential proangiogenic effects of IL-33 (19, 68). Yeon-Sook Choi et al. demonstrated that IL-33 increases proliferation and differentiation of human endothelial cells and increases angiogenesis *in vivo* (68). We previously demonstrated increased densities of IL-33 positive and ST2 positive microvessels in adenoma/CRC stroma, suggesting that microvessels are not only the cellular sources of IL-33 and ST2 may also be autocrine or paracrine targets for IL-33/ST2 in regulating angiogenesis in the adenoma/CRC microenvironment (19). IL-33 can stimulate the production of pro-angiogenic factors (77, 78), vascular endothelial growth factor (VEGF), and IL-8, are well-known pro-angiogenic factors in human cancers including CRC (18, 79). There is evidence to show that IL-33 strongly stimulates production of VEGF and IL-8 *in vitro* (77, 78). We and others have reported significant increase of IL-8 and VEGF expression in human adenoma and CRC specimens (18, 19, 80), which are similar to the expression profile of IL-33 in adenoma and CRC specimens. Therefore, these findings may suggest a possible regulatory effect of IL-33 on the induction of pro-angiogenic factors during colorectal tumorigenesis.

Induced suppressive immune cell accumulation: Numerous studies have demonstrated that IL-33 can greatly influence various immune cells during differentiation, immune responses and homeostasis (45, 65). Thus, one of the possible mechanisms for IL-33 in promoting human cancer progression is through increased accumulation of immunosuppressive cells within the tumor microenvironment and by diminishing innate anti-tumor immunity. The IL-33/ST2 axis has a potential role in the expansion and function of diverse immune cells (65). Accumulation of Tregs in the tumor microenvironment is a common phenomenon in both colorectal premalignant and malignant lesions (8, 81, 82). IL-33 can induce the enhanced recruitment of suppressive immune cells such as CD11b+ GR1+ and CD11b+ F4/80+ myeloid cells and Tregs into the tumor site, where these cells have a strong impact on immune microenvironment remodeling (60, 83). Exogenously administered recombinant mouse IL-33 significantly potentiates tumor burden and induces ST2 positive Tregs infiltrated into tumor masses. In contrast, neutralizing IL-33 or ST2 by administration of antibodies remarkably inhibits tumor size and decreases the density of ST2 positive Tregs inside tumor masses in tumor-bearing mice (84). Thus, the accumulation of suppressive immune cells in the tumor microenvironment might play a role in the regulation of host immune response during CRC development.

**Figure 1:**
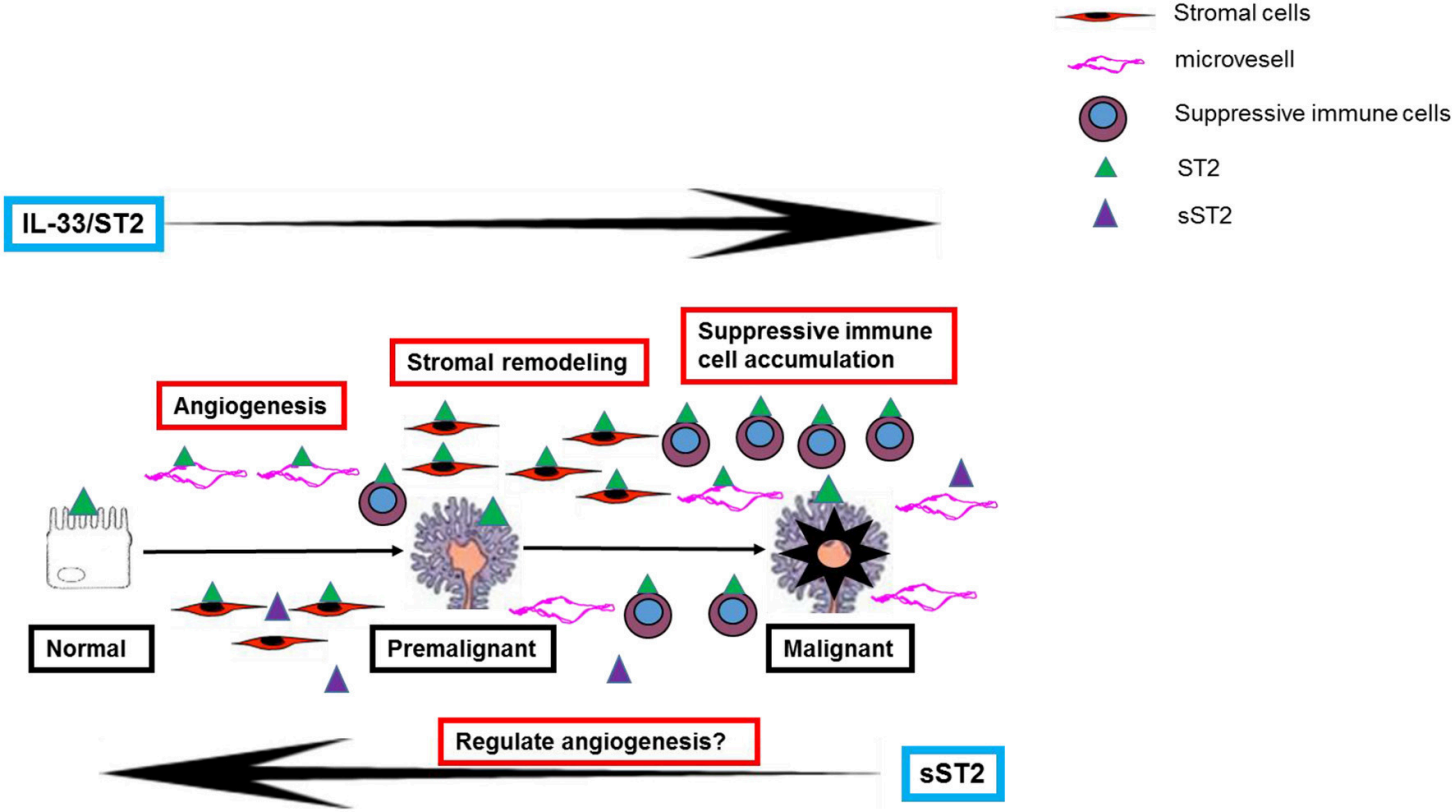
A schematic representation of the potential mechanisms of IL-33 in contributing to the development of colorectal premalignant and malignant lesions.

Therefore, although the exact mechanism of IL-33's promotion of CRC development is still unknown, its pro-tumor function in the colorectal mucosa is likely due to a combination of several mechanisms (see Figure 1).

## Could IL-33 be a potential biomarker and new therapeutic target for colorectal tumorigenesis?

It was previously demonstrated that IL-33 and ST2 at mRNA and protein levels are increased in both premalignant and malignant tissues (19, 63, 85). In our study, we investigated the clinical significance of IL-33 and ST2 as novel clinicopathological biomarkers in patients with premalignant adenomas and CRCs. General analysis revealed that no clinicopathological parameters were associated with tissue levels of either IL-33 or ST2 mRNAs in patients with adenomas. However, tissue expression levels of ST2 in CRC patients were associated with TNM stage, and patients with advanced TNM stage had a higher ST2 mRNA level than those with early stage disease (19). It has been established that the expression level of IL-33 is associated with poor prognosis and predicts unresponsiveness to chemotherapy in certain cancers, including CRC (55, 56, 58, 86). We therefore analyzed the relationship between expression levels of IL-33/ST2 and overall survival in patients with CRCs. However, our Kaplan-Meier analysis did not confirm a correlation between concentrations of either IL-33 or ST2 at the mRNA level with overall survival times in CRC patients (19). These results indicate that whether high expression levels of IL-33 and ST2 mRNA could serve as a marker of disease aggressiveness in patients with CRC is still questionable. IL-33 and ST2 are expressed in a variety of cells, including epithelial cells, stromal cells, infiltrating lymphocytes and microvessels (19), and IL-33 and ST2 at the mRNA level of whole CRC specimens quantified by real-time PCR may not differentiate the expression level of IL-33 and ST2 in various compartments of the tumor microenvironment. Therefore, a future priority is to investigate the relationship between expression levels of IL-33 and ST2 in various compartments and clinicopathological variables.

Many efforts have been directed toward the inhibition of cytokine networks as new therapeutic targets for CRC development (87). The IL-33/ST2 axis as a new therapeutic target during the CRC development has also been evaluated. One study to show that the blockade of ST2 signaling through the administration of a ST2 antibody suppresses the development of CRC in ApcMin/+ mice (59). Similarly, ST2 deficiency reduces tumor number, size, and dysplastic grade in the intestine of AOM/DSS-dosed mice (63, 88).

Because angiogenesis is a critical step for the growth, expansion and metastasis of CRCs, targeting angiogenesis has become one of the therapeutics for treating CRCs (87). As we stated previously, IL-33 is a potent pro-angiogenic factor that markedly enhances angiogenesis during tumor development. We and others have previously demonstrated that both IL-33 and ST2 are highly expressed in tumor-associated microvessels, implying autocrine stimulation of angiogenesis during the progression of CRC (19). Cytokine signaling can be easily targeted pharmacologically (89), and several studies have examined the inhibitory efficacy of IL-33 or ST2 signals in murine CRC models. However, cytokine networks in tumors are very complex; one cytokine may have a wide range of biological effects, while different cytokines may possess the same function (38). Although most studies demonstrate that IL-33 promotes the development of CRC, the antitumor effect of IL-33 has also been reported at least in some tumor models (90–92). Therefore, one should take into consideration that IL-33 might have a more complex effect on the development of CRC when designing therapeutic interventions targeting IL-33 or ST2 signaling. Fundamental questions regarding the mechanisms of IL-33 or ST2 signal blockade in preventing the development of CRC remain to be further investigated.

## Conclusions and future perspectives

Cumulative evidence from both animal and human studies strongly supports that IL-33 plays an important role in dictating the development and growth of colorectal cancer through regulation of host anti-tumor immunity, angiogenesis and stromal remodeling that are found in the tumor microenvironment. Based on the findings of the studies outlined in this review, IL-33 as a potential contributor and therapeutic target for the development of colorectal tumorigenesis has been considered, which may benefit the design of new medicines for the treatment of CRC. However, many fundamental questions remain.

## Author contributions

GC and AY offered direction on the project and drafted the manuscript. ZP, WZ, ZL, and RG reviewed the manuscript and made significant revisions to the manuscript. All authors read and approved the final manuscript.

### Conflict of interest statement

The authors declare that the research was conducted in the absence of any commercial or financial relationships that could be construed as a potential conflict of interest.

## Abbreviations

CRC: colorectal cancer
IL: interleukin
Treg: regulatory T cell
IR: immunoreactivity
IBD: inflammatory bowel diseases
COX-2: cyclooxygenase-2
IEC: intestinal epithelial cell
NF-κB: Nuclear Factor-κB
AOM: azoxymethane (AOM)
DSS: dextran sodium sulfate.
